# Closure versus nonclosure of buccal mucosal graft harvest site: A prospective randomized study on post operative morbidity

**DOI:** 10.4103/0970-1591.45541

**Published:** 2009

**Authors:** K. Muruganandam, Deepak Dubey, Anil Kumar Gulia, Anil Mandhani, Aneesh Srivastava, Rakesh Kapoor, Anant Kumar

**Affiliations:** Department of Urology, Sanjay Gandhi Postgraduate Institute of Medical Sciences, Rae Bareli Road, Lucknow, India

**Keywords:** Buccal mucosa harvest, post operative morbidity

## Abstract

**Objective:**

To prospectively compare the postoperative morbidity of closure versus non closure of the buccal mucosal graft (BMG) harvest site.

**Methods:**

Patients who underwent BMG harvest for urethroplasty were randomized into 2 groups; in group 1 donor site was closed and in group 2 it was left open. Self made questionnaires were used to assess post-operative pain, limitation to mouth opening, loss of sensation at graft site. The time to resumption of liquid and solid diet were also noted.

**Results:**

Fifty patients were studied, 25 in each group from July 2003 to July 2005. BMG was harvested from single cheek in most of the patients. Mean post operative pain score was 4.20 and 3.08 at day 1 in group 1 and group 2, respectively (*P* < 0.05). Return to oral intake in terms of liquid and solid diet was comparable between the groups. Difficulty with mouth opening was maximal during the first week with no difference among the two groups. Two patients in group 1 and one in group 2 had persistent peri-oral numbness at 6 months. None of the patients in both the groups had changes in salivation or retention cysts.

**Conclusion:**

Pain appears to be worse in the immediate post operative period with suturing of the harvest site. There is no difference in long term morbidity whether the graft site is closed or left open. It may be best to leave buccal mucosa harvest sites unsutured.

## INTRODUCTION

After the initial report by Burger *et al* in 1992,[1] buccal mucosal graft (BMG) has become popular for reconstruction of anterior urethra with acceptable post operative outcomes.[2–4] BMG can be easily harvested from the inner cheeks or lower lip with minimal morbidity.[5] There are only few studies with small number of patients which report about the oral complications[6–9] and only one series in the current literature which prospectively studied the effect of closure or leaving the graft site open on post operative morbidity.[10] We have conducted a prospective randomized study to compare the postoperative morbidity of closure versus non closure of the BMG harvest site in patients undergoing BMG urethroplasty.

## MATERIALS AND METHODS

Patients undergoing BMG substitution urethroplasty were randomized in to two groups depending upon whether the graft harvest site was closed (group 1) or left open (group 2). The method of randomization was every alternate patient (1:1 ratio) undergoing buccal mucosal harvesting assigned to group 1 or 2. Demographic patient profile, stricture related characteristics like etiology, site, length of stricture and type of surgery performed were noted. Buccal mucosa was harvested from inner cheeks and lower lip depending upon the length required.

### Technique of bmg harvest

Naso-tracheal intubation is ensured and a retractor is used for keeping the mouth wide open. The parotid duct opening is identified and protected. The submucosal plane is infiltrated with 1% xylocaine and adrenalin solution (1 in 100,000). The site is marked with a scalpel, stay stitches were taken on buccal mucosa and then the flap of buccal mucosa is dissected from underlying buccinator muscle with fine scissor. After the graft is harvested, perfect hemostasis is achieved with bipolar electrocautery and an adrenalin soaked gauge piece is left over the harvest site for about 4 to 6 h.

Graft harvest site was either closed (group 1) or left open (group 2) depending on which group the patient was assigned to. In group 1, patients the mucosal edges were approximated with continuous interlocking sutures using 3 ‘O’ vicryl. Patients were given self made questioners to assess pain, mouth opening and loss of sensation at graft site. Pain score was recorded for initial 5 days once daily using visual analog score (see Appendix). Return to liquids, solid diet and any salivatory difficulty in post operative period starting from day 1 were also noted. Same parameters were noted at follow up at 3 and 6 months. Statistical analysis was done using non parametric Mann Whitney test and a *P* value of less than 0.05 was considered as significant.

## RESULTS

A total of 50 patients (25 in each group) were studied, from July 2003 to July 2005, with a mean age of 35.2 years (range 17-72) in group 1 and 35 (range 18-64) years in group 2. The distribution of etiology of stricture was similar among both the groups [Table 1]. The number of patients with panurethral stricture was more in group 2 (7 versus 1 in group 1) and the mean stricture length was also significantly more in group 2 as compared to group 1 i.e., 8.36 ± 1.96 cm (range 2-17 cm) and 4.84 ± 1.49 cm (range 3-10 cm), respectively. Correspondingly, the area of the harvested graft was significantly more in group 2 as compared to group 1 (14.9 cm^2^ vs 8.6 cm^2^) [Table 1]. Buccal mucosa was most commonly harvested from the single cheek i.e., 19/25 and 15/25 patients in groups 1 and 2, respectively. Six patients in group 2 and none from group 1 had buccal graft harvested from the lower lip.

**Table 1:** Patient and stricture characteristics

Characteristics	Group 1 (*n*=25)	Group 2 (*n*=25)	*P* value
Age in years (mean) (range)	35.2 (17–72)	35.4 (18–64)	NS
Stricture etiology	Inflammatory - 7	Inflammatory - 13	
	Traumatic - 6	Traumatic - 6	0.15
	Iatrogenic - 12	Iatrogenic - 6	
Site of stricture	Panurethral - 1	Panurethral - 7	
	Panbulbar - 1	Panbulbar - 2	0.117
	Proximal bulb - 10	Proximal bulb - 6	
	Distal bulb - 7	Distal bulb - 6	
	Penobulbar - 6	Penobulbar - 4	
Length of stricture (cm)	4.84 (3 10)	8.36 (2–17)	<0.05
Area of the graft (cm^2^)	8.6 (5.3–18)	14.9 (3.6–30.6)	<0.05

**Figure 1:**
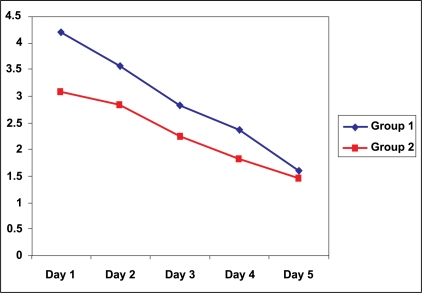
Comparison of pain scores between group 1 (closed) and 2 (not closed)

The post operative morbidities after the buccal graft harvesting are shown in Table 2. All patients had maximum pain at the first post operative day with pain score falling more promptly in group 2 as compared to group 1 on subsequent days [Figure 1]. None of the patients in either group had significant pain at 3 months and 6 months post operatively.

**Table 2:** Post operative morbidity after buccal mucosal graft harvest

Parameter	Group 1 (*n*=25)	Group 2 (*n*=25)	*P* value
Pain score			
At day 1	4.20 ± 0.71	3.08 ± 0.95	0.004
Day 2	3.56 ± 0.51	2.84 ± 0.80	.004
At day 5	1.60 ± 0.50	1.44 ± 0.51	0.09
At 6 months	1.08	1.04	NS
No. of patients with difficult mouth opening			
Immediate Post-operative	22	24	NS
At 1 week	2	4	0.06
At 6 months	0	2	0.06
No. of patients with perioral numbness			
Immediate Post-operative	6	4	NS
At 1 week	3	1	
At 6 months	2	1	
No. of patients tolerating liquid diet at day 1	23	24	NS
No. of patients tolerating solid diet at day 3	20	23	
Salivatory problems	3	0	
Retention cysts	1	0	

The majority of patients in both groups had difficulty in opening the mouth at post operative day one (22 patients in group 1 and 24 patients in group 2) which improved at one week post operative (only two patients in group 1 and four patients in group 2 had difficulty in mouth opening at 1 week). At six months post operative, only two patients in group 2 had difficulty in mouth opening. Both these patients had undergone 1^st^ stage urethroplasty and buccal mucosa was harvested from both cheeks and lower lip for first stage repair. None of the patients from group 1 had similar problem.

Six patients in group 1 and four patients in group 2 complained perioral numbness at post operative day 1 which had improved significantly at one week post operatively. Only three patients in group 1 and one patient in group 2 had perioral numbness at one week. The perioral numbness persisted in two patients from group 1 and one patient from group 2 at six months.

**Figure 2:**
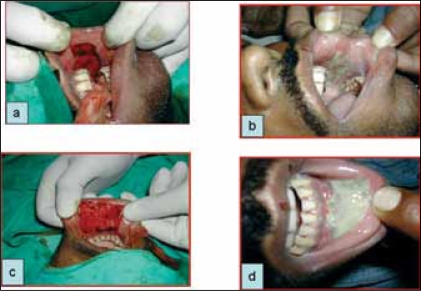
Buccal musoca donor site on post operative day 0 and 3 for cheek mucosa (a and b) and lower lip mucosa (c and d), respectively

Donor site of all patients in group 2 showed excellent healing on post operative day 3 [Figure 2]. Almost all the patients were tolerating liquids at post operative day 1 (23 patients in group 1 and 24 patients in group 2) and majority of patients could tolerate solid diet at post operative day 3 (20 patients in group 1 and 23 patients in group 2).

Three patients in group 1 had transient salivatory problems on ipsilateral side and one patient had retention cyst which resolved spontaneously at six months where as none of the patients in group 2 had any similar complaints. Mean hospital stay was 4.2 days in both the groups. None of these patients from either group had significant bleeding, hematoma or wound healing problems.

## DISCUSSION

Buccal mucosa urethroplasty has been more popular in the last few years, after recognition of its feasibility and very good outcome as well as its low morbidity at the reconstruction site.[4] Advantages of buccal mucosa as a free graft are that it is hairless, has a thick elastin rich epithelium making it tough and easy to handle and also having thin and highly vascular lamina propria that facilitates inosculation and imbibition.[11,12] Buccal mucosa is most commonly harvested from the cheek, unilateral or bilateral, depending upon the length of graft required. Cheek is the preferred site in most of the studies as it provides wide and long grafts.[8–10] An alternative to cheek is mucosa from the lower lip but its width limits the size of the graft, so it is used along with cheek mucosa when required length is more. Kamp *et al*, reported that harvesting the buccal graft from the lower lip resulted in a significantly greater long-term morbidity, which resulted in a lower proportion of satisfied patients. This seems to be due to a long-lasting neuropathy of the mental nerve.[7] Closure of lip mucosa may also lead to eversion of vermilion and lip contracture. In our series, mucosa from the lower lip was used in six cases, all of which required graft length more than 13 cm and harvest site was left open in all six cases. There were no cosmetic deformities or long-term morbidity noted.

Conventionally the donor area used to be closed after the graft harvesting mainly because of concerns about the hemostasis and adequate healing of the raw area. Closure of the donor area may result in increased pain due to the stretching of the mucosal edges and poor cosmesis especially in lower lip. There is only one prospective study which has reported the effect of non closure of graft harvest site on post operative morbidity compared with a group of patients in which graft harvest site was closed.[10]

In our prospective randomized study, pain was the most common symptom in the post operative period and was maximal at first post operative day in both the groups. Mean pain score was significantly higher in the group 1 in which donor site was closed i.e., 3.5 as compared to 2.7 in group 2. This is in spite of the fact that the mean area of the graft harvested in group 2 was significantly more than in group 1 and six patients in group 2 had the graft harvested from lower lip also. This shows that not suturing the donor area leads to lesser pain whether larger grafts were harvested or graft harvested from lower lip. In the prospective study by Wood *et al*, the mean pain score for patients with donor site closure was significantly higher than that for patients without donor site closure (*P*< 0.01).[10] Patient in whom donor site is closed may complain of more pain due to tight approximating sutures.

Restriction of mouth opening was most bothersome in the first week after surgery. It was seen in almost all the patients and resolved completely by three weeks except for two patients in group 2 who had persistent problems at six months. Similar observations have been made by others as in one series, 12 out of 14 patients had initial difficulty with mouth opening which resolved completely by 3 months.[6] Dublin *et al*, reported that 32% patients undergoing buccal mucosal harvesting had restriction of mouth opening at the end of 20 months, where the buccal mucosal harvest site was closed in all patients.[8] In our study, only two patients in group 2 and none from group 1 had difficulty in mouth opening at 6 months. Both patients in group 2 who had persistent problem with mouth opening at 6 months had panurethral strictures and underwent buccal mucosal harvest from both the cheeks and lower lip.

Perioral numbness is related to a reduction in sensation in the region of the graft harvest and is an unavoidable consequence of excision of mucosa. In our study, six patients in group 1 and 4 patients in group 2 complained transient perioral numbness which had improved in majority of the patients at 1 week and only 2 patients from group 1 and 1 patient from group 2 had persistent perioral numbness at 6 months. No patient suffered any damage to nerves and no change in sensation along nerve territories was reported. Dublin *et al* from a retrospective study of 30 patients (all patients had donor area closed), reported that 16% of patients had oral numbness for a mean duration of 13.6 months.[8]

Immediate resumption to liquid diet was seen in all except one patient in group 1. Eighty percent of patients in group 1 and 93% patients in group 2 were able to resume normal diet by the end of third post operative day. Similar observations have been reported by others.[8,10]

In our study, the complications like salivatory problems, mucous retention cysts occurred in very few patients transiently which resolved spontaneously at 6 months. Similarly in the study reported by Wood *et al*, 11% of patients reported changes in salivation and 2% had mucous retention cyst that required excision.[10]

Other problems related to graft harvest like hematoma at harvest site, persistent bleeding requiring revisit to operation theatre or packing or any wound healing problems were not seen in our series. It shows that once perfect hemostasis is achieved the donor area can be left open without causing additional problems.

## CONCLUSION

Pain appears to be worse in the immediate post operative period after suturing the harvest site. There is no difference in long-term post operative morbidity whether the graft site is closed or left open. It may be best to leave buccal mucosa harvest sites unsutured.

## APPENDIX 1: QUESTIONNAIRE ASSESSING PAIN

**Table:**

Point	Degree of pain
1.	No pain
2.	Minimal
3.	Moderate
4.	Severe
5.	Unbearable
